# Improvements in the Diagnostic Performance of Coronary Computed Tomography Angiography in the Assessment of Heavily Calcified Plaques Using a Novel Deep Learning Model

**DOI:** 10.31083/RCM53504

**Published:** 2026-09-14

**Authors:** Zhonghua Sun

**Affiliations:** ^1^Discipline of Medical Radiation Sciences, Curtin School of Diagnostic and Therapeutic Sciences, Curtin University, 6102 Perth, Australia; ^2^Curtin Medical Research Institute (Curtin MRI), Curtin University, 6102 Perth, Australia

Coronary computed tomography angiography (CCTA) is a widely accepted imaging modality for the routine diagnosis of coronary artery disease (CAD) and has significantly improved patient management, as demonstrated by large multicentre studies [1,2,3,4,5]. With continued technological advancements in computed tomography (CT) imaging, particularly the recent introduction of photon-counting CT, CCTA has achieved high diagnostic accuracy in patients with suspected CAD [6,7,8]. Despite these advances, one of the major challenges compromising the diagnostic performance of CCTA is the presence of extensive or heavy coronary artery calcification. High-density calcium produces blooming artifacts that obscure the coronary lumen, resulting in overestimation of luminal stenosis, leading to high false-positive rates. This limitation results in reduced specificity and positive predictive value (PPV) [9,10]. Clinically, these false-positive findings often lead to unnecessary downstream investigations, particularly invasive coronary angiography (ICA), which could be avoided in patients without hemodynamically significant coronary stenosis.

The limitations of CCTA in the evaluation of calcified coronary plaques have been extensively investigated. Numerous strategies have been proposed to mitigate the impact of calcium-related blooming artifacts. These approaches include advanced image post-processing and subtraction techniques [11,12], the application of iterative reconstruction algorithms [13,14], improvements in spatial resolution (**Supplementary Fig. 1**) [6,7,8,15], and the use of deep learning (DL)–based methods specifically designed to suppress blooming artifacts (Fig. 1, Ref. [16]) [16,17,18,19]. Overall, these studies have demonstrated moderate improvements in the specificity and PPV of CCTA compared with assessment of conventional lumen-based stenosis.

**Fig. 1.**
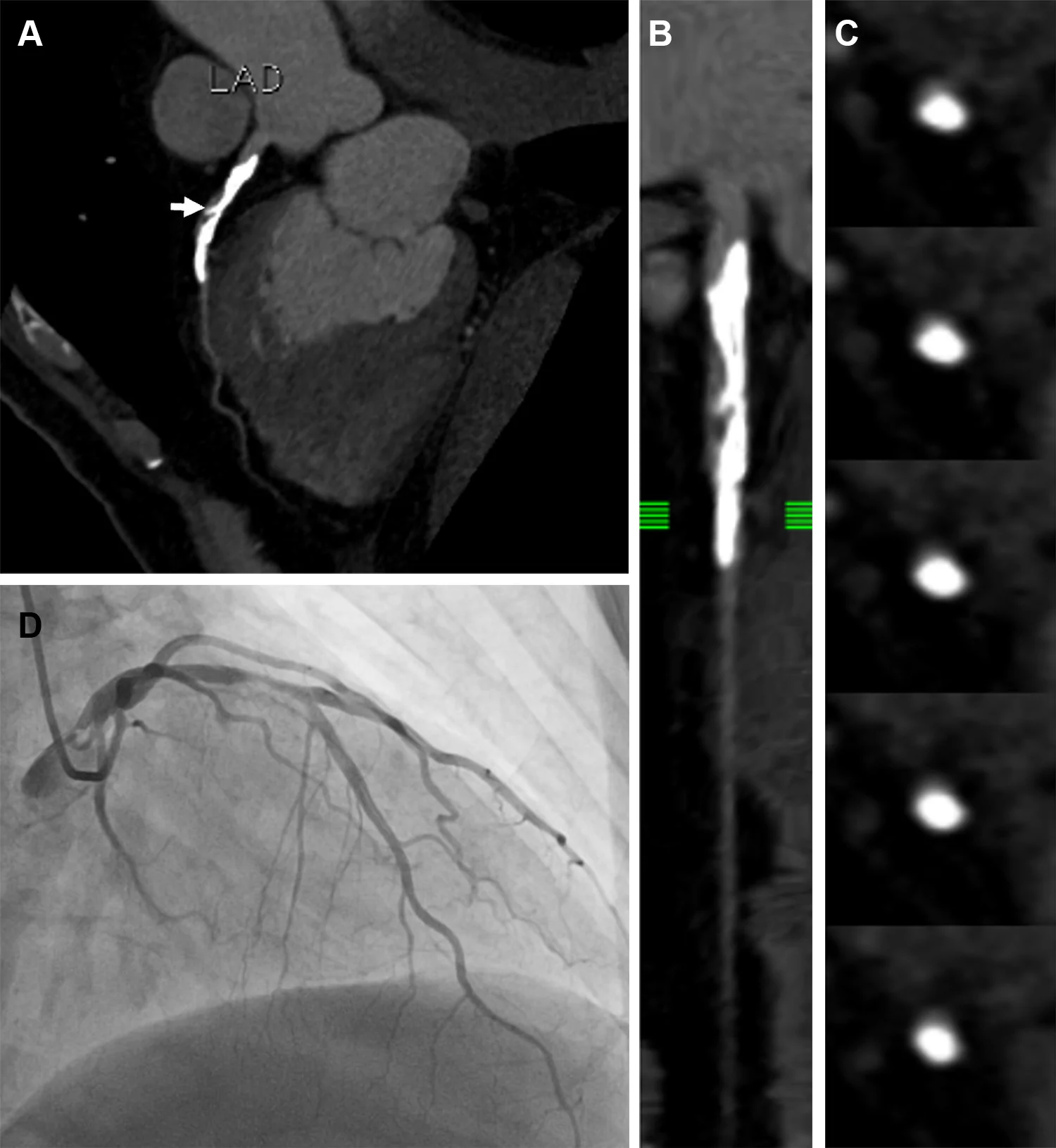
**Sample of the true-positive case of deep learning in diagnosing obstructive stenosis (≥50%)**. (A–C) Luminal stenosis was 70%–99% on curved planar reconstruction and transverse view by a deep learning model. The green lines in the 1B indicate cross sections at 1-mm intervals. (D) Luminal stenosis was more than 70% at quantitative coronary angiography. LAD, left anterior descending. Arrow in 1A refers to the calcified plaque. Reprinted with permission under open-access licence from Wang et al. [16].

Despite these advances, existing strategies are constrained by several important limitations. Many proposed methods are confined to research settings and lack widespread clinical implementation, while others rely on advanced imaging technologies that are not readily available in routine clinical practice in many centers. In addition, a number of studies are limited by small sample sizes, which restricts their ability to demonstrate robust improvements in diagnostic performance. Consequently, although false-positive rates have been reduced, satisfactory specificity and PPV have not yet been consistently achieved.

Therefore, there remains an urgent need to develop more advanced and clinically applicable approaches to further enhance the diagnostic accuracy of CCTA in the presence of heavily calcified coronary plaques. This critical research gap has recently been addressed by a study reported by Shi et al. [20].

In a recent issue of *Reviews in Cardiovascular Medicine*, Shi et al. [20] developed a novel self-supervised DL algorithm and evaluated its performance in 786 consecutive patients who underwent both CCTA and ICA. This multicentre study involved four hospitals in China, with patients randomly allocated to a training cohort (80% of the dataset, *n* = 628) and a validation cohort (20%, *n* = 158). All patients with severe coronary calcification (Agatston score >300) were included, reflecting the study’s specific focus on improving the assessment of coronary lumen stenosis in the presence of heavy calcification.

CCTA examinations were performed using 256-slice and 128-slice CT scanners. Image interpretation was conducted independently by two experienced radiologists, and their assessments were compared with ICA as the reference standard to determine diagnostic performance. These results were then further compared with DL-processed CCTA images. In addition to evaluating luminal stenosis, defined as a ≥50% reduction in diameter, the authors also quantified the extent of coronary calcification using cross-sectional calcium arc measurements, categorized as minimal (<90°), mild (90°–180°), moderate (180°–270°), and severe (270°–360°) calcification.

The proposed DL model, termed a multi-granularity masked autoencoder (MGMAE), differs from previous DL-based plaque characterization approaches in that it was specifically designed to optimize the assessment of coronary lumen stenosis in heavily calcified segments. The MGMAE framework integrates both global and local mask autoencoders, with the global component preserving overall vessel course and anatomical context, while the local component focuses on calcified segments where lumen boundaries are obscured. This approach enables effective separation of calcification from the coronary lumen and surrounding structures, thereby mitigating the impact of calcium-related blooming artifacts. The overall workflow of the DL algorithm implemented in this study is illustrated in Fig. 2 (Ref. [20]).

**Fig. 2.**
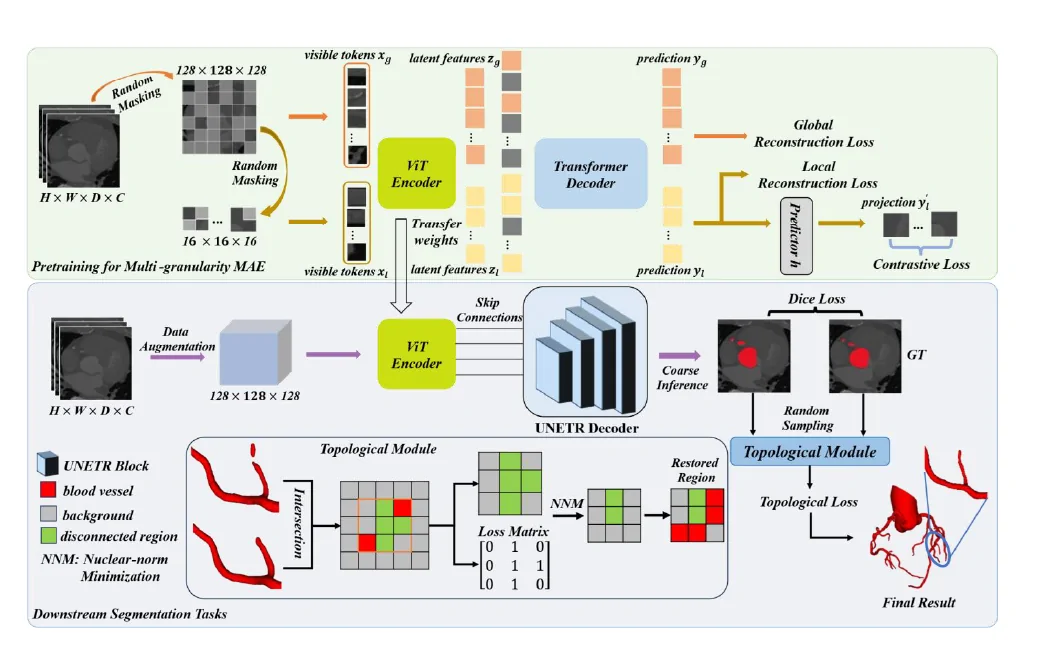
**Framework of the MGMAE for self-supervised coronary artery segmentation**. During the pre-training stage, a global masked autoencoder (G-MAE) and a local MAE (L-MAE) are employed for image reconstruction, combined with contrastive learning to guide local diversity representation. In the fine-tuning stage, the pre-trained encoder is utilized to support downstream coronary artery segmentation, with an imposed topological constraint to preserve coronary anatomical structure. MGMAE, multi-granularity masked autoencoder; G-MAE, global masked autoencoder; L-MAE, local masked autoencoder. Reprinted with permission under open-access licence from Shi et al. [20]. NNM, nuclear normal maximization.

The results demonstrated the robustness of the proposed model. When evaluated on the training dataset using the MGMAE framework, the model achieved a dice similarity coefficient (DSC) of 92.3% and a Hausdorff distance of 1.28 mm, indicating excellent lumen segmentation performance. At the per-lesion, per-vessel, and per-patient levels, the model achieved sensitivities and specificities of 78% and 51%, 83% and 52%, and 84% and 74%%, respectively. In the validation cohort of 158 patients, a total of 1090 calcified plaques from 632 coronary arteries were evaluated. Among these, 476 calcified plaques in 213 coronary arteries from 93 patients were confirmed to have ≥50% luminal stenosis by ICA. Across all assessment levels, the DL model outperformed the radiologist-based CCTA interpretation, with statistically significant differences observed at the per-lesion, per-vessel, and per-patient levels (all *p* < 0.01).

Importantly, the DL model led to substantial improvements in specificity and PPV. At the per-vessel level, specificity and PPV increased from 30% and 37% based on radiologists’ interpretation to 52% and 47% with DL-assisted assessment. Similarly, at the per-patient level, specificity and PPV improved from 57% and 72% to 74% and 82%, respectively.

When stratified by the severity of calcification, the DL model consistently improved diagnostic performance, and its advantage was most pronounced in severely calcified lesions. In plaques with cross-sectional calcium arcs of 180°–270° and 270°–360°, sensitivity, PPV, and negative predictive value (NPV) increased from 32%/45%/51%, 12%/55%/43%, to 50%/57%/71%, and 39%/65%/79%, respectively (all *p* < 0.01), clearly demonstrating the model’s superiority over a conventional radiologist’s assessment in heavily calcified segments.

Furthermore, benchmark comparisons against two state-of-the-art DL models, UNEt TRansformers (UNETR) and MAE3D, revealed significantly improved DSC and Hausdorff Distance values for the MGMAE model (all *p* < 0.001), underscoring its superior capability in accurately delineating the coronary lumen despite severe calcification. In addition to diagnostic gains, the DL approach markedly reduced reporting time and decreased interpretation time from 376 s for radiologist-based assessment to 61.9 s with the DL model.

Two important findings from Shi’s study [20] warrant further discussion. First, the study evaluated the developed DL model in a relatively large, multicentre cohort, which substantially strengthens the validity and generalizability of the findings. This represents an important contribution to the existing literature, as most previous studies investigating strategies to improve the diagnostic performance of CCTA in the presence of calcified plaques were limited by small sample sizes and single-center designs [10,11,13,15,17,18,19]. In recent years, DL algorithms have been increasingly applied in cardiovascular imaging, including automatic coronary artery calcium detection, plaque identification and quantification, and prediction of disease outcomes [16,21,22,23,24,25,26]. Although many of these studies have demonstrated moderate to high diagnostic performance, they primarily focused on reporting internal model accuracy without conducting direct comparisons with existing DL approaches. In contrast, Shi’s study [20] directly compared the performance of their DL model with that of other comparable DL algorithms for the assessment of calcified plaques. This comparative evaluation provides more robust evidence supporting the superiority of the proposed DL model over a radiologist’s interpretation and previously reported artificial intelligence (AI)-based methods.

Second, an additional contribution of this study lies in the detailed analysis of the extent of calcified plaques and its influence on CCTA diagnostic performance across different calcium-arc groups. The proposed DL model demonstrated marked improvements in diagnostic accuracy, increasing specificity, PPV, and NPV by up to 36 percentage points compared with a conventional radiologist’s interpretation. In most previous studies, the severity of calcified plaques was defined primarily using the Agatston score (typically >300), without further stratification of plaque distribution. In contrast, Shi’s study [20] performed a more granular assessment by analyzing the cross-sectional calcium arc in addition to the calcium score. Notably, the most significant diagnostic improvements were observed in lesions with extensive calcification, where blooming artifacts most severely compromise the evaluation of the lumen. This finding has important clinical implications, as reducing false-positive CCTA interpretations in heavily calcified plaques may help avoid unnecessary downstream invasive investigations and improve patient management.

In summary, Shi and colleagues [20] developed a novel DL algorithm and evaluated its performance in the assessment of more than 1000 calcified coronary plaques, demonstrating significant improvements in specificity and positive predictive value compared with a radiologist’s interpretation. The DL model showed particular advantages over human readers in the assessment of extensively calcified plaques. However, despite these encouraging results obtained from a relatively large dataset, the overall diagnostic value of the model remains low to moderate, as the specificity and positive predictive value were below 60% at both the per-lesion and per-vessel levels. This limitation was particularly evident in plaques with extensive calcification (calcium arc 270°–360°), where specificity reached only 39%, although this still represented a substantial improvement compared with the markedly low sensitivity of 12% achieved by radiologists. This indicates that over 60% of hemodynamically significant stenoses caused by heavy calcification would be missed. The authors will need to explicitly discuss their model’s applicability at the current performance level and suggest further improvements to increase its sensitivity. Since the CCTA data were acquired from multiple centers using different generations of scanners, the image quality could affect their model performance. Therefore, the authors should consider a subgroup analysis or covariate adjustment. These findings highlight the need for further refinement and optimization of the DL model to achieve more robust improvements in diagnostic performance, particularly in heavily calcified lesions.
