# Supplementary material for: Improvements in the Diagnostic Performance of Coronary Computed Tomography Angiography in the Assessment of Heavily Calcified Plaques Using a Novel Deep Learning Model
Source: Rev Cardiovasc Med. 2026 Sep 14;27(9):53504. doi: 10.31083/RCM53504 (PMC13653167; doi:10.31083/RCM53504)
Supplement: Supplementary file 1 [file 2153-8174-27-9-53504-s1.zip › Supplementary Fig. 1.docx]

**Supplementary Fig. 1. Representative coronary computed tomography (CT) angiography images of the right coronary artery (RCA) from the same patient acquired using (A) energy-integrating detector CT (EID-CT) and (B) photon-counting CT.** Compared with EID-CT, photon-counting CT provides improved visualization of coronary artery calcifications due to its superior spatial resolution and reduced calcium-related blooming artifacts. Reprinted with permission under open access from Nakashima et al. [1].

References

[1] Nakashima M, Miyoshi T, Hara S, Miyagi R, Nishihara T, Miki T, et al. Photon-Counting CT Enhances Diagnostic Accuracy in Stable Coronary Artery Disease: A Comparative Study with Conventional CT. Journal of Clinical Medicine. 2025; 14: 6049. https://doi.org/10.3390/jcm14176049
